# PraeKids: Diagnoseprävalenz lebensbedrohlicher und lebensverkürzender Erkrankungen bei Kindern und Jugendlichen in Deutschland

**DOI:** 10.1007/s00103-023-03704-5

**Published:** 2023-06-07

**Authors:** Nadja Melina Burgio, Sven Jennessen

**Affiliations:** Abteilung Pädagogik bei Beeinträchtigungen der körperlich-motorischen Entwicklung, Institut für Rehabilitationswissenschaften, Kultur‑, Sozial- und Bildungswissenschaftliche Fakultät, Humboldt-Universität zu Berlin, Unter den Linden 6, 10099 Berlin, Deutschland

**Keywords:** Lebenslimitierende Erkrankung, Pädiatrie, Palliative Care, Palliativmedizin, Diagnoseschlüssel, Life-limiting illness, Pediatrics, Palliative care, Palliative medicine, Diagnosis code

## Abstract

**Einleitung:**

In Deutschland wird bis dato von einer Prävalenz von ca. 50.000 Kindern und Jugendlichen ausgegangen, die mit lebensbedrohlichen und lebensverkürzenden Erkrankungen leben. Diese in der Versorgungslandschaft kommunizierte Zahl beruht auf einer Übertragung empirischer Daten aus England.

**Methoden:**

In Zusammenarbeit mit dem Spitzenverband Bund der Krankenkassen (GKV-SV) und dem Institut für angewandte Gesundheitsforschung Berlin GmbH (InGef) wurden die Abrechnungsdaten der von den gesetzlichen Krankenkassen dokumentierten spezifischen Behandlungsdiagnosen der Jahre 2014–2019 analysiert und erstmals Prävalenzdaten von Betroffenen im Alter von 0–19 Jahren erhoben. Zudem wurden mittels der Daten von InGef die Prävalenzwerte nach Diagnosegruppierung, den *Together-for-Short-Lives(TfSL)-Gruppen 1–4,* und auf Grundlage der in den englischen Prävalenzstudien verwendeten (und hier aktualisierten) Kodierungsliste berechnet.

**Ergebnisse:**

Die Datenanalyse ermöglichte die Festlegung eines Prävalenzbereichs von 319.948 (InGef – adaptierte Fraser-Liste) bis 402.058 (GKV-SV) unter Berücksichtigung der TfSL-Gruppen. Die TfSL-1-Gruppe stellt mit 190.865 Erkrankten die größte Gruppe dar.

**Diskussion und Fazit:**

Erstmalig liegen durch diese Untersuchung für Deutschland Prävalenzwerte von 0‑ bis 19-Jährigen mit lebensbedrohlichen und lebensverkürzenden Diagnosen vor. Da sich im Forschungsdesign die Falldefinitionen und die einbezogenen Versorgungssettings (ambulant/stationär) unterscheiden, differieren die aus den Daten des GKV-SV und des InGef erhobenen Prävalenzwerte. Aufgrund der sehr heterogenen Krankheitsverläufe, Überlebenschancen und Mortalitätsraten können keine unmittelbaren Ableitungen auf palliative und hospizliche Versorgungsstrukturen getroffen werden.

## Abstract (en)

**Introduction:**

In Germany, a prevalence of approximately 50,000 children and adolescents living with life-threatening and life-limiting diseases is currently assumed. This number, which is communicated in the supply landscape, is based on a simple transfer of empirical data from England.

**Methods:**

In cooperation with the German National Association of Health Insurance Funds (GKV-SV) and the Institute for Applied Health Research Berlin GmbH (InGef), the billing data of the specific treatment diagnoses documented by statutory health insurance funds for the years 2014–2019 were analyzed and – for the first time – prevalence data of affected 0‑ to 19-year-olds were collected. In addition, the data from InGef was used to calculate the prevalence by diagnosis grouping, the *Together for Short Lives (TfSL) groups 1–4,* and based on the (updated) coding lists from the English prevalence studies.

**Results:**

The data analysis determined a prevalence range of 319,948 (InGef – adapted Fraser list) to 402,058 (GKV-SV), with consideration of the TfSL groups. The TfSL‑1 group represents the largest group with 190,865 patients.

**Discussion and conclusion:**

This study is the first to provide the prevalence of 0‑ to 19-year-olds living with life-threatening or life-limiting diseases in Germany. Since the case definitions and the included care settings (outpatient/inpatient) differ in the research design, the prevalence values collected from the GKV-SV and InGef are also different. Due to the very heterogeneous course of the diseases, chances of survival, and mortality rates, no direct conclusions can be drawn for palliative and hospice care structures.

## Einleitung

In Deutschland wird bis dato von ca. 50.000 Kindern und Jugendlichen im Alter von 0–19 Jahren ausgegangen, die mit lebensbedrohlichen und lebensverkürzenden Erkrankungen leben [1–3]. Dieser Wert basiert auf Ergebnissen von Prävalenzstudien von Fraser et al. aus England [4], die auf Deutschland übertragen und hochgerechnet wurden [2]. Da die englischen Studien Patient:innen bis zur Vollendung des 19. Lebensjahres berücksichtigen und diese Daten die Grundlage für die in der deutschen Palliativversorgung verwendeten Prävalenzwerte von Kindern und Jugendlichen darstellen [1, 2], fokussiert die vorliegende PraeKids-Studie^1^ ebenfalls diesen Altersbereich mit dem Ziel der internationalen Vergleichbarkeit der Daten.

Lebensbedrohliche und lebensverkürzende Diagnosen können in unterschiedliche Erkrankungsgruppen, die sogenannten *Together-for-Short-Lives(TfSL)-Gruppen 1–4,* eingeteilt werden. Komprimiert handelt es sich dabei um

- *TfSL-1:* lebensbedrohliche Erkrankungen, für die eine kurative Therapie verfügbar ist, die jedoch auch versagen kann (z. B. Tumorerkrankungen),
- *TfSL-2:* Erkrankungen, die nicht kurativ behandelbar sind, für die jedoch lebensverlängernde Therapieoptionen bestehen (z. B. Mukoviszidose),
- *TfSL- 3:* progrediente Erkrankungen ohne die Möglichkeit lebensverlängernder, sondern ausschließlich palliativer Therapie (z. B. Adrenoleukodystrophie),
- *TfSL-4:* irreversible, meist neurologische, jedoch nicht progrediente Erkrankungen, die wahrscheinlich zu einem vorzeitigen Tod führen (z. B. hypoxische Enzephalopathie; [5, 6]).

## Methoden

Das Projekt PraeKids gliederte sich in 2 Stufen. In der ersten Projektstufe wurden im Rahmen qualitativer Expert:inneninterviews Relevanz, bisherige Zugangswege und Optionen valider Datenerhebungen zur Ermittlung der Prävalenz der Zielgruppe eruiert. Dafür wurden nationale und internationale Interviewpartner:innen aus verschiedenen Zuständigkeitsbereichen für lebensbedrohliche und lebensverkürzende Erkrankungen im Kindes- und Jugendalter kontaktiert und mit 7 von ihnen Leitfadeninterviews geführt. Der Fokus der Interviews lag auf forschungsmethodischen Optionen zur Datenerhebung und Prävalenzberechnung. Durch die Analyse der Interviewdaten wurde deutlich, dass die Expert:innen die Erhebung deutscher Prävalenzzahlen für absolut notwendig und wichtig erachten. Allerdings zeigte sich auch, dass Wege der Datenerhebung bislang weder bekannt waren, noch dass es diesbezüglich Untersuchungen gab. Es wurde daher festgelegt, die englischen Erhebungen zur Orientierung zu nutzen, indem der Zugang zur Prävalenz über die Kodierungen der ICD-10 (Internationale statistische Klassifikation der Krankheiten und verwandter Gesundheitsprobleme) erfolgen sollte. Entsprechend wurde mit Beginn der zweiten Projektstufe ein Studiendesign für die Prävalenzerhebung konzipiert, verschiedene Institutionen bzgl. verfügbarer Optionen der Datenerhebung kontaktiert und schließlich der Spitzenverband Bund der Krankenkassen (GKV-SV) und das Institut für angewandte Gesundheitsforschung Berlin GmbH (InGef) für eine Zusammenarbeit und die Aufbereitung der spezifischen Krankenkassendaten akquiriert.

Um festzulegen, welche Diagnosen in die Prävalenzberechnung einfließen sollen, wurde eine Liste mit Diagnosekodierungen erstellt. Als Grundlage diente hierfür die ICD-10-Liste nach Fraser et al. [4], mittels welcher auch die Prävalenzen in England und Schottland [7–9] erhoben worden waren. Die in dieser Liste genutzten ICD-10-Codes wurden zunächst durch 4 Palliativmediziner:innen hinsichtlich der zentralen Kriterien der Lebensbedrohung und Lebensverkürzung bewertet, wobei von den Mediziner:innen teils unterschiedliche Codes ein- bzw. ausgeschlossen wurden. Aufgrund dieser differierenden Einschätzungen wurden im nächsten Schritt 3 Mediziner:innen zu einer Online-Gruppendiskussion eingeladen, in der nach fachlichen Kriterien entschieden wurde, ob die als kritisch gewerteten Codes (u. a. Kloakenpersistenz, die Kurzripp-Polydaktylie-Syndrome und die Refsum-Krankheit) bei der Prävalenzerhebung berücksichtigt werden sollten. Neben der fachlichen Einschätzung von Lebensbedrohung oder Lebensverkürzung durch die Erkrankung war auch die Zuordnung zu einer TfSL-Gruppe Ziel des Fachaustauschs. Diese Kategorisierung ist jedoch aufgrund der sich ständig weiterentwickelnden Therapiemöglichkeiten als dynamisch zu betrachten und bildet deshalb immer nur den zum jeweiligen Bearbeitungsstand aktuellen Stand ab. Tab. 1 zeigt die Diagnosen, die von den Palliativmediziner:innen als weder lebensbedrohlich noch lebensverkürzend eingestuft und entsprechend aus der Prävalenzberechnung ausgeschlossen wurden.

**Table 1:** 

Kodierung	Titel
E34.8	Sonstige näher bezeichnete endokrine Störungen
E88.1	Lipodystrophie, andernorts nicht klassifiziert
E80.3	Erworbene Aphasie mit Epilepsie (Landau-Käfer-Syndrom)
G60.0	Hereditäre sensomotorische Neuropathie
H11.1	Konjunktivadegeneration und -einlagerungen
H35.5	Hereditäre Netzhautdystrophie
Q39.6	Ösophagusdivertikel (angeboren)
Q74.8	Sonstige näher bezeichnete angeborene Fehlbildungen der Extremität(en)
Q77.4	Achondroplasie
Q78.5	Metaphysäre Dysplasie
Q87.1	Angeborene Fehlbildungssyndrome, die vorwiegend mit Kleinwuchs einhergehen
Q87.2	Angeborene Fehlbildungssyndrome mit vorwiegender Beteiligung der Extremitäten

In Vorbereitung auf die Gruppendiskussion wurde zudem die Liste mit den ICD-10-Codes auf Aktualisierungen und Änderungen überprüft, da die von Fraser et al. [4] genutzte Liste ausschließlich ICD-10-Codes aus dem Jahr 2011 enthielt. Hierbei wurden zusätzliche Kodierungen aus der aktuellen ICD-10-Liste identifiziert, die als lebensbedrohlich und/oder lebensverkürzend eingestuft werden könnten, jedoch nicht in den englischen Studien [4, 10, 11] berücksichtigt wurden. Auch diese wurden im Rahmen der Gruppendiskussion auf Lebensbedrohung und Lebensverkürzung sowie die TfSL-Gruppierung bewertet. Es entstand eine Liste mit aktuellen ICD-10-Codes (Burgio-Jennessen-Liste), die sowohl Codes von Fraser et al. [4] als auch neu hinzugefügte Diagnosekodierungen enthielt.^2^ Diese Liste ist die Grundlage für die Erhebung der Prävalenz anhand der Krankenkassendaten der GKV-SV und InGef [12]. Zudem wurde die Fraser-Liste modifiziert, in dem die als nicht lebensbedrohlich bzw. lebensverkürzend eingestuften Diagnosen entfernt wurden. Diese Liste dient im Studienverlauf lediglich zum Datenabgleich.

### Prävalenzwerte auf Grundlage der GKV-SV-Daten

Für die Prävalenzwerterhebung wurde eine Sekundärdatenanalyse mittels der Routinedaten des GKV-SV durchgeführt. Diese stellen eine wichtige Grundlage der Versorgungsforschung und Qualitätssicherung im Gesundheitswesen dar [13, 14]. Im Jahr 2020 waren rund 73 Mio. Menschen (etwa 88 % der Bevölkerung) in der Bundesrepublik Mitglied in der gesetzlichen Krankenversicherung (GKV; [15]).

Für die Übermittlung der Diagnoseprävalenz nutzt der GKV-SV nach Angaben des Stabsbereichs Vertragsanalyse einen Auszug aus den Versichertenstammdaten und kollektivvertraglichen Abrechnungsdaten der gesetzlich Versicherten. Es ist eine Stichprobe, die für jedes Datenjahr auf einer wechselnden Basis von 7 Geburtskalendertagen erhoben wird. Die Hochrechnung auf die gesamte GKV erfolgt anhand der amtlichen GKV-Versichertenzahlstatistik KM 6, welche zum Stichtag 01.07. eines Jahres erhoben wird und auf der Internetseite des Bundesgesundheitsministeriums^3^ verfügbar ist. Die KM 6 ist unterteilt in 17 KV-Bezirke und 34 Alters- und Geschlechtsgruppen. Die Hochrechnungsfaktoren werden jährlich neu bestimmt, da von Jahr zu Jahr ein Kalendertag neu hinzukommt und ein vorheriger wegfällt. Zudem verändert sich die Versichertenzahl in der GKV gesamt von Jahr zu Jahr.

Für die Berechnung der Prävalenzzahl laut GKV-SV wurde jede versicherte Person mit einer Diagnose aus der Burgio-Jennessen-Liste einmal gezählt, auch wenn weitere lebensbedrohliche oder lebensverkürzende Diagnosen gestellt wurden. Die Prävalenzwerte des GKV-SV wurden auf die Bevölkerung hochgerechnet, in dem der Prozentanteil der GKV-Versicherten mit mindestens einer der Erkrankungen mit der Anzahl der Bevölkerung im Alter von 0–19 Jahren [16] multipliziert wurde. Weitere Gewichtungen nach Region oder Alters- und Geschlechtsgruppen wurden dabei nicht vorgenommen.

Die stationären Diagnoseprävalenzen sind nicht abbildbar, da der GKV-SV in diesem Versorgungsbereich nur Diagnosefälle und nicht die Versicherten zählen kann und somit kein dem Forschungsinteresse entsprechendes Merkmal in der Datenbank zur Verfügung steht.

### Prävalenzwerte auf Grundlage der InGef-Daten

InGef verfügt über eine Datenbank anonymisierter Abrechnungsdaten von rund 6 Mio. gesetzlich Versicherten aus etwa 60 Betriebs- und Innungskrankenkassen [17, 18]. Dies entspricht einer Teilmenge der Krankenkassen, die auch durch den GKV-SV erfasst werden. In der Datenbank befinden sich Daten der letzten 6 Jahre – beginnend mit dem aktuellen vollständigen Jahr. Bei der Analyse der InGef-Datenbank zeigt sich, dass die Daten der am 01.01.2009 Versicherten (*N* = 6.197.045) bis zum 31.12.2013 zu 78,5 % in der Datenbank blieben und somit kein Wechsel der Krankenversicherung stattfand [18]. Für die aktuelle Erhebung kann entsprechend davon ausgegangen werden, dass auch die über ihre Eltern Versicherten mit lebensbedrohlichen und/oder lebensverkürzenden Erkrankungen zu einem großen Anteil nicht in eine andere Krankenkasse wechselten.

Die Prävalenzerhebung durch InGef erfolgte über die Jahre 2014–2019 im Sinne einer Querschnittsanalyse. Es galten folgende Einschlusskriterien:

- vollständige Versicherung vom 01.01. bzw. ab Geburt bis zum 31.12. bzw. bis zum Tod innerhalb des jeweiligen Analysejahres,
- Alter von maximal 19 Jahren am 31.12. des jeweiligen Analysejahres.

**Figure 1:**
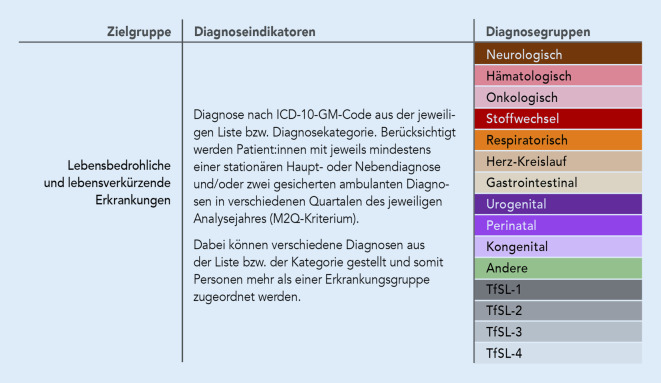

Hinsichtlich der Falldefinition wurden Patient:innen mit jeweils mindestens einer stationären Haupt- oder Nebendiagnose und/oder 2 gesicherten ambulanten Diagnosen in verschiedenen Quartalen des jeweiligen Analysejahres (M2Q-Kriterium) berücksichtigt. Dabei können grundsätzlich alle Diagnosen aus der Burgio-Jennessen-Liste gestellt werden. Die spezifische Zuordnung der Erkrankungen aus dieser Liste erfolgt dann in den anderen Falldefinitionen (Abb. 1). Ebenso sollte die Prävalenz anhand der ICD-10-Codes der modifizierten Fraser-Liste erhoben werden. Abb. 1 gibt einen Überblick über die Falldefinitionen.

Zudem wurden als weitere Variablen festgelegt:

- *Alter* am 31.12. des jeweiligen Analysejahres, eingeteilt in die Altersgruppen < 1 Jahr, 1 bis < 2 Jahre, 2 bis < 3 Jahre, 3 bis < 6 Jahre, 6 bis < 10 Jahre, 10 bis < 15 Jahre, 15 bis < 19 Jahre und 19 bis < 20 Jahre,
- *Geschlecht* (männlich/weiblich) am 31.12. des jeweiligen Analysejahres,
- Wohnort nach* Bundesland* am 31.12. des jeweiligen Analysejahres.

Die Prävalenz lebensbedrohlicher und lebensverkürzender Erkrankungen in ihren jeweiligen Kategorien (siehe Falldefinitionen) wurde für die einzelnen Jahre 2014–2019 berechnet. Jede Kategorie wurde dabei separat betrachtet. Als Bezugsbevölkerung galten alle Personen aus der InGef-Datenbank, die die Einschlusskriterien erfüllen.

## Ergebnisse

### Prävalenzwerte auf Grundlage der GKV-SV-Daten

**Figure 2:**
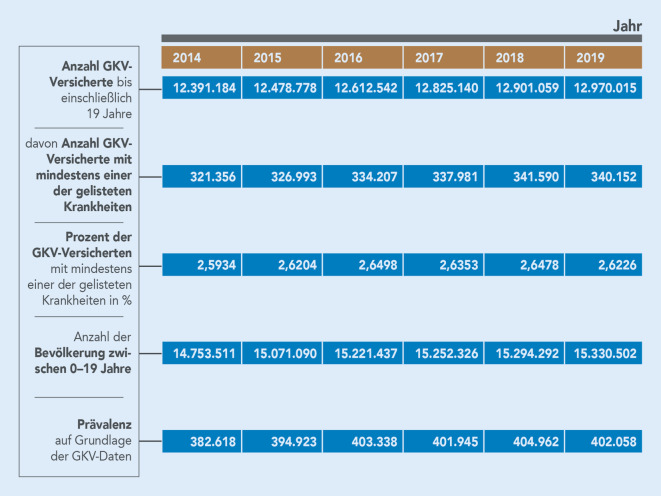

In Abb. 2 werden die Prävalenzwerte aus den Jahren 2014–2019 des GKV-SV und deren Hochrechnung auf die Bevölkerung dargestellt. Es handelt sich um Daten aus der ambulanten Versorgung.

### Prävalenzwerte auf Grundlage der InGef-Daten

**Figure 3:**
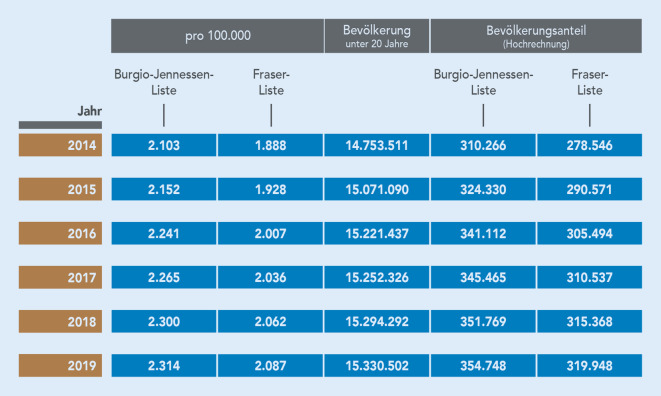

Auch die durch InGef ermittelten Prävalenzwerte aus den Jahren 2014–2019 wurden auf die Bevölkerung hochgerechnet (Abb. 3). Zudem werden zum Vergleich auch die Werte anhand der adaptierten Fraser-Liste präsentiert.

### Prävalenzrange

Es wird ersichtlich, dass unter Verwendung sowohl der Burgio-Jennessen-Liste als auch der adaptierten Fraser-Liste in den Datensätzen des GKV-SV und von InGef eine deutlich höhere Prävalenz von Kindern und Jugendlichen mit lebensbedrohlichen und/oder lebensverkürzenden Erkrankungen aufgezeigt wird als der in der Literatur verwendete Transferwert von 50.000 Betroffenen [2, 3].

Die InGef-Daten wurden des Weiteren in diagnostische Gruppen aufgeschlüsselt. Es zeigt sich, dass kongenitale Erkrankungen die größte Gruppe darstellen, gefolgt von Stoffwechsel‑, Krebs- und respiratorischen Erkrankungen. Die kleinste Diagnosegruppe besteht aus lebensbedrohlichen oder lebensverkürzenden gastroenterologischen Erkrankungen. Bei der Erfassung der Werte ist bedeutsam, dass aufgrund von Komorbiditäten insgesamt mehr Diagnosen erfasst werden, als der Prävalenzwert abbildet. Es ist festzuhalten, dass in den Jahren 2014–2019 eine Zunahme der Diagnosestellungen in allen Gruppen zu verzeichnen ist. Lediglich die onkologischen Diagnosen verzeichnen einen leichten Rückgang (2014: 313 pro 100.000 Betroffene; 2019: 301 pro 100.000 Betroffene).^4^

Bei der Stratifizierung nach Altersgruppen zeigt sich, dass im Jahr 2019 die häufigsten Diagnosestellungen bei den < 1-Jährigen vorgenommen wurden und die geringsten bei den 6‑ bis < 10-Jährigen. Bei männlichen Kindern und Jugendlichen kam es in den Jahren 2014–2019 zu einer Zunahme der Diagnosen von 2215 auf 2432 pro 100.000 und bei den weiblichen von 1986 auf 2189 pro 100.000 Personen. Die nach Bundesländern stratifizierte Diagnoseprävalenz zeigt die häufigste Diagnosestellung lebensbedrohlicher und/oder lebensverkürzender Erkrankungen in Bremen, gefolgt von Sachsen-Anhalt.

Bei den Diagnoseprävalenzen nach den TfSL-Gruppen 1–4 finden sich auf die Bevölkerung hochgerechnet in der *Gruppe 1* 190.865 Kinder und Jugendliche mit Erkrankungen aus der aktuell erstellten ICD-10-Liste^5^ nach Burgio-Jennessen, in der *Gruppe 2* 127.243, in der *Gruppe 3 *22.076 und in der *Gruppe 4* 56.263 Kinder und Jugendliche.

**Figure 4:**
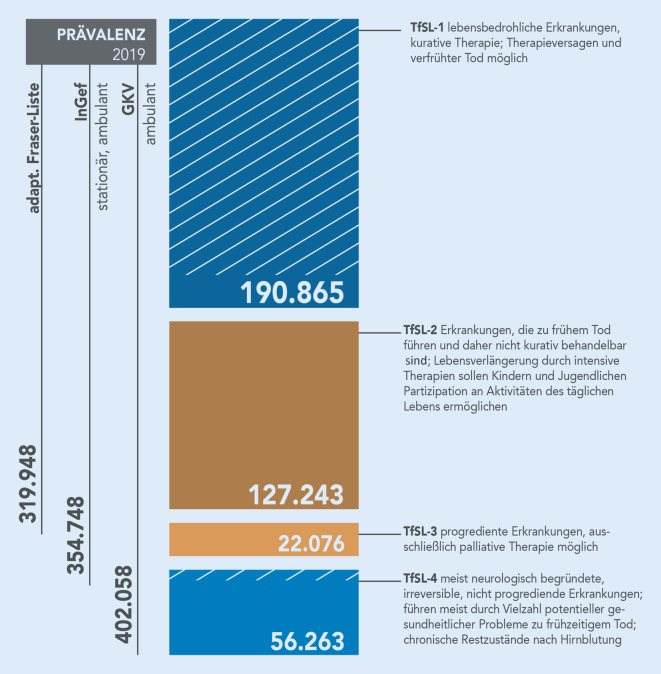

Auf der Grundlage dieser Datenquellen und Berechnungen wurden die Ergebnisse des GKV-SV und InGef auf Basis der Burgio-Jennessen-Liste in einer Prävalenzrange zusammengefasst. Diese liegt zwischen 319.948 (InGef, adaptierte Fraser-Liste) und 402.058 (GKV-SV; Abb. 4). Die gestreiften Markierungen in der Darstellung der TfSL-Gruppen 1 und 4 zeigen den Anteil erkrankter Kinder und Jugendlicher auf, die prognostisch überleben bzw. keiner palliativer Versorgung und hospizlicher Begleitung bedürfen.

## Diskussion

In der aktuellen Untersuchung wurde eine Sekundärdatenanalyse durchgeführt. Der große Vorteil lag darin, dass dadurch Personen erfasst werden konnten, die aufgrund ihrer lebensbedrohlichen und lebensverkürzenden Erkrankungen vermutlich nicht für eine Primärdatenerhebung zur Verfügung gestanden hätten [16]. Des Weiteren entfällt bei dieser Form der Analyse ein Drop-out oder eine Verweigerung zur Teilnahme und da es sich um Routinedaten handelt, werden Verzerrungen wie Erinnerungs- und Interviewbias vermieden [16–19].

Die PraeKids-Studie zeigt einen deutlichen Unterschied zwischen der erhobenen Prävalenzzahl von über 300.000 diagnostizierten Betroffenen und der in der Literatur verwendeten, jedoch nicht empirisch erhobenen Anzahl von 50.000 Kindern und Jugendlichen mit lebensbedrohlichen und lebensverkürzenden Erkrankungen. Die Studie von Fraser et al. [4], auf die sich die letztgenannte Zahl bezieht, und die vorliegende Untersuchung beruhen auf unterschiedlichen Forschungsdesigns inklusive divergierender Falldefinitionen. Bei PraeKids wurden im Gegensatz zu den englischen Studien sowohl stationäre als auch ambulante Fälle einbezogen (sofern letztere das M2Q-Kriterium erfüllten), da nicht alle Kinder und Jugendlichen mit lebensbedrohlichen Gesundheitsverläufen regelmäßiger stationärer Versorgung bedürfen. Des Weiteren wurden in der Prävalenzberechnung aus Deutschland auch lebensbedrohliche Erkrankungen berücksichtigt, die nicht zwingend zu einem frühen Tod und einer palliativen Versorgung führen müssen.^6^ Sie können aber einer akuten (intensiv-)medizinischen bzw. -pflegerischen Versorgung bedürfen und/oder sollten je nach Behandlungsverlauf zumindest potenziell in der zukünftigen Versorgung mitbedacht werden, auch wenn sie im günstigsten Fall (erfolgreiche kurative Therapie) keine dauerhaft intensive sowie palliative Versorgung benötigen. Ein Vergleich der Daten mit weiteren aktuellen Studien ist aufgrund der unterschiedlichen Methodiken oder Zielsetzungen nicht möglich [21, 22].

Zudem sind kleinere Unterschiede in der Kodierungspraxis zu berücksichtigen: In der Zusammenarbeit mit den Palliativmediziner:innen aus Deutschland gab es lediglich bei 5 % der Diagnosen divergierende Einschätzungen bezüglich des Ein- bzw. Ausschlusses von ICD-10-Codes in die aktuelle Prävalenzberechnung. Hierbei wurde auf der Basis von fachlich-medizinischen Einschätzungen über die Berücksichtigung dieser ICD-10-Codes entschieden.

Die in der Literatur und der Versorgungslandschaft verwendete Zahl von 50.000 basiert auf einer Hochrechnung, die allerdings unterschiedliche internationale Bevölkerungs- und Versorgungsmerkmale sowie Sterberaten nicht berücksichtigt. So zeigen Analysen, dass Deutschland eine rund 25 % geringere Kindersterblichkeit bei den unter 5‑Jährigen aufweist als Großbritannien [23]. Diese unterschiedlichen Sterberaten und Bevölkerungsmerkmale wie der Anteil von Menschen aus Einwandererfamilien haben möglicherweise auch Einfluss auf unterschiedliche Prävalenzraten in der PraeKids-Studie und bei Fraser et al. [11]. In England stieg die Prävalenz von 2013/2014 zu 2018 um ca. 17,7 %, in Deutschland von 2014 zu 2018 um ca. 9,2 % [12]. Die Unterschiede lassen sich möglicherweise auch mit unterschiedlichen Kodierpraxen, länderspezifischen Abrechnungsmodalitäten und der Zunahme eines gewachsenen Wissensstandes bezüglich der Erkrankungen bei den kodierenden Ärzt:innen begründen.

### Prävalenzwerte auf Grundlage der Daten von GKV-SV und InGef

Die Unterschiede in den errechneten Prävalenzwerten zwischen dem GKV-SV und InGef liegen an den abweichenden Falldefinitionen und kassenbedingten Unterschieden der Versichertengruppen. Entsprechend ist der niedrigere Prävalenzwert von InGef durch das differenziertere Einschlussverfahren begründet. Während hier nämlich mindestens eine stationär und/oder 2 ambulant gestellte Diagnosen in verschiedenen Quartalen des Erhebungszeitraums berücksichtigt wurden, war bei dem GKV-SV eine ambulante Diagnoseerfassung aus der Burgio-Jennessen-Liste ausreichend. Es war aufseiten des GKV-SV nicht möglich, die engeren Falldefinitionen an die von InGef anzugleichen, und wie bereits erläutert, konnten auch die dokumentierten stationären Fälle des GKV-SV aufgrund des fehlenden Merkmals in der Datenbank nicht berücksichtigt werden.

Durch den beschriebenen generellen Ausschluss des Geburtskalendertags 1 in der GKV-SV-Datenerhebung kann es zu Selektionseffekten kommen. Insbesondere Personen aus Einwandererfamilien werden nach Aussagen des Stabsbereichs Vertragsanalyse dadurch nicht im vollen Umfang erfasst. Die Ursache ist, dass im Rahmen des Aufnahmeprozesses das Geburtsdatum, falls es nicht bekannt oder dokumentiert ist, oft willkürlich auf den ersten Tag eines Monats gelegt wird. Für Personen aus Einwandererfamilien ist überdurchschnittlich oft der erste Monatstag als Geburtstag angegeben, wodurch sie vom Ausschluss des ersten Geburtskalendertags überproportional häufig betroffen sind.^7^ Diese vom GKV-SV genannten möglichen Selektionseffekte wurden insofern für die PraeKids-Studie vernachlässigt, da es sich nicht um die Erhebung eines absoluten Prävalenzwertes handelt, sondern um die Festlegung einer Range. Es kann somit davon ausgegangen werden, dass die minimalen Auswirkungen durch die Festlegung eines Prävalenzbereiches Berücksichtigung finden.

Bezüglich der onkologischen Prävalenz fällt der leichte Rückgang in den Jahren 2014–2019 auf. Das Deutsche Kinderkrebsregister meldet hier eine Zunahme der Erkrankungen von 2051 im Jahr 2013/2014 auf 2255 Erkrankungen im Jahr 2019 [24]. Damit stehen diese Zahlen im Widerspruch zu den aktuell erhobenen onkologischen Prävalenzwerten durch InGef. Diese Datenunterschiede können zum einen damit in Zusammenhang stehen, dass im Kinderkrebsregister Jugendliche unter 18 Jahren erfasst werden, während die PraeKids-Daten entsprechend der britischen Studie Patient:innen bis einschließlich des 19. Lebensjahres berücksichtigen. Zum anderen zeigen die Werte aus den Jahren 2014–2019 überschneidende Konfidenzintervalle, sodass der beschriebene Rückgang statistisch betrachtet vernachlässigt werden kann.

### Limitationen der Studie

Die ermittelten Prävalenzwerte weisen gewisse Limitationen auf. Sie basieren auf Abrechnungsdaten der dokumentierten stationären und ambulanten Diagnosen, die klinisch nicht überprüfbar und somit in ihrer Validität eingeschränkt sind [25]. Die Festlegung auf ausgewählte ICD-10-Codes und damit die Auseinandersetzung mit Einzeldiagnosen, Diagnosegruppen, der entsprechenden Kategorisierung in lebensbedrohliche und lebensverkürzende Erkrankungen und der Zuordnung in die TfSL-Gruppen kann, wie bereits beschrieben, aus fachlicher Perspektive unterschiedlich erfolgen. Hier dienten die oben beschriebenen Rückkopplungsschleifen mit den kooperierenden Mediziner:innen einem Höchstmaß dialogischer Validität. Am Beispiel des ICD-10-Codes E74 *„*Sonstige Störungen des Kohlenhydratstoffwechsels“ soll eine mit der Datengrundlage verbundene Herausforderung dargestellt werden. So wurde dieser Code mit in die Prävalenzberechnung einbezogen, da hereditäre Störungen des Galaktose- oder Fruktosestoffwechsels zu lebensbedrohlichen Krisen in der Neugeborenenperiode führen können [26]. Eine eindeutige Abgrenzung dieser Diagnosen von beispielsweise der nicht lebensbedrohlichen intestinalen Fruktoseintoleranz kann auf Grundlage des ICD-10-Schlüssels nicht vorgenommen werden. Hier ermöglicht die ICD-10-Systematik – wie auch bei vielen seltenen Erkrankungen – keine weitere Differenzierung. Ein weiteres Beispiel für die Kodierungsimponderabilität stellt der Code J96 „Respiratorische Insuffizienz“ dar. Im Jahr 2020 fielen laut des GKV-SV auf

- J96.0 „Akute respiratorische Insuffizienz, anderenorts nicht klassifiziert“ 4460 Diagnosen,
- J96.1 „Chronische respiratorische Insuffizienz, anderenorts nicht klassifiziert“ 2392 Diagnosen,
- J96.9 „Respiratorische Insuffizienz, nicht näher bezeichnet“ 6314 Diagnosen.

Auch hier ist eine differenzierte Auswertung nicht möglich. Ursachen sind Struktur und fehlende Differenziertheit der ICD-10 als klassifikatorisches Dokumentationssystem. Symptome, Syndrome und Krankheitsbilder können nicht segmentierter kategorisiert, sondern teils nur zusammenfassend erfasst werden [27]. In Absprache mit den an der vorliegenden Untersuchung beteiligten Palliativmediziner:innen wurden trotz dieser Limitation die teils als durchaus kritisch zu bewertenden Codes mit in die Untersuchung aufgenommen.

Bezüglich der Diagnosestellung spielen auch die Abrechnungsmodalitäten über diagnosebezogene Fallgruppen (DRG) und das damit einhergehende Upcoding^8^ hinsichtlich der Abrechnungsrelevanz und leistungsorientierten Vergütung eine Rolle. Hier kann es zu Überdokumentationen kommen, durch die die Hauptentlassungsdiagnose nicht als die ursächliche Indikation für den stationären Aufenthalt erfasst wird [27]. Die geplante Digitalisierung abrechnungstechnisch relevanter Daten könnte dieses Problem zukünftig reduzieren.

Im Projekt PraeKids fanden Rückkopplungsschleifen mit Expert:innen statt. So wurden stichprobenartige Überprüfungen der Einstufungen in die TfSL-Gruppen nach der Gruppendiskussion erneut durch Mediziner:innen durchgeführt und auf Übereinstimmung geprüft. Die Ergebnisse wurden zudem durch einen erfahrenen Versorgungsforscher gesichtet, sodass insgesamt von einer hohen inhaltlichen Validität ausgegangen werden kann. Allerdings war eine Validitätsprüfung durch die Vergleiche von Stichproben im Rahmen einer internen und externen Validierung nicht möglich [27].

Zudem muss bei der Einteilung in die TfSL-Gruppen berücksichtigt werden, dass einzelne Diagnosen im Krankheitsverlauf unterschiedlich zugeordnet werden können, d. h., ein zu Beginn lebensbedrohlicher Zustand (TfSL-1) kann in einen chronischen Restzustand (TfSL-4) übergehen. Des Weiteren ist bei den Ergebnissen zu konstatieren, dass eine einfache Angleichung an die Größe der Grundgesamtheit vorgenommen wurde und somit weitere Randbedingungen wie zusätzliche soziodemografische Verteilungseigenschaften in der Stichprobe sowie der Grundgesamtheit nicht beachtet wurden. Dies steht in Zusammenhang mit den zur Verfügung gestellten Daten. Die Sekundärdaten des GKV-SV und von InGef enthalten keine Informationen zu den Lebensbedingungen der Patient:innen. Diese werden teils nicht miterhoben bzw. können aufgrund von Datenschutzbestimmungen nicht genutzt werden.

Zudem wurden die vorliegenden Prävalenzzahlen aus Daten der GKV der unter 20-Jährigen errechnet. Die Zahlen der Privaten Krankenversicherung (PKV) blieben unberücksichtigt. Im Jahr 2020 waren 1,56 Mio. 0‑ bis 19-jährige Personen privat krankenversichert.^9^ Da die Prävalenz auf Grundlage von ca. 13 Mio. GKV-Versicherten erhoben wurde, hat die Anzahl der PKV-Versicherten eine statistisch nur geringfügige Auswirkung auf die Prävalenz. Inwieweit es zu minimalen Verschiebungen des Prävalenzwertes aufgrund der beispielsweise sozioökonomischen Struktur der PKV-Versicherten kommen würde, wäre in Folgeuntersuchungen genauer zu betrachten [20].

Des Weiteren können keine Aussagen über Entscheidungskriterien bei der klinischen Diagnosestellung und der Dokumentation gemacht werden, da die Erhebung mittels Sekundärdaten erfolgte, wobei von einer gewissen Varianz bezüglich der ambulanten und stationären Diagnosen ausgegangen werden kann [28, 29]. Der ambulante Bereich kodiert quartalsweise, der stationäre Bereich dokumentiert die Aufnahme‑, Einweisungs- und Entlassungsdiagnosen. Für die Abrechnung erfolgt entsprechend bei der Entlassung die Feststellung einer Hauptdiagnose und fakultativer Nebendiagnosen [30]. Es kann also von einer entsprechenden Varianz in der Diagnosestellung ausgegangen werden, die sich beispielsweise in unterschiedlichen Kodierungen zu Beginn der Aufnahme und der Entlassung des Kindes zeigen kann. Die daraus resultierenden Kodierungswechsel konnten in der Studie nicht berücksichtigt werden, da ausschließlich die Entlassungsdiagnose bzw. die zuletzt gestellte Diagnose aus dem Abrechnungsquartal einbezogen wurde.

## Fazit

Die aus empirischen Studienergebnissen lediglich abgeleitete Anzahl von 50.000 Kindern und Jugendlichen mit lebensbedrohlichen und lebensverkürzenden Erkrankungen in Deutschland bildet die Grundlage für die derzeitigen Versorgungsstrukturen sowie gesundheits- und bildungspolitische Entscheidungen in Bezug auf die Zielgruppe. Allerdings zeigen die in der vorliegenden Arbeit präsentierten Daten, dass die Diagnosezahl der Kinder und Jugendlichen mit lebensbedrohlichen und lebensverkürzenden Erkrankungen in den Jahren 2014–2019 weit über diesem Wert liegt. Der berechnete Prävalenzbereich bildet nicht nur die Gesamtprävalenz im ambulanten und stationären Setting ab, sondern berücksichtigt auch die einzelnen TfSL-Gruppen, die für die Gestaltung von Versorgungsstrukturen aufgrund der Unterscheidung in Lebensbedrohung und Lebensverkürzung eine wichtige Basis bilden.

Aus den Ergebnissen lässt sich nicht unmittelbar ein quantitativer Versorgungsbedarf der Zielgruppe ableiten. Hierfür bedarf es zusätzlicher Daten zur Morbidität und zu den Bedarfen der Kinder und Jugendlichen sowie ihrer Familien in verschiedenen *Phases of Illness* [31] in Bezug auf die derzeitige Angebotsstruktur der einzelnen Bundesländer. Zudem ist bei einem Versorgungsabgleich auch die Diversität der Krankheitsverläufe und der daraus abgeleiteten Bedarfe an medizinisch-pflegerischer Versorgung sowie psychosozialer Begleitung und Beratung zu berücksichtigen.

Es bleibt festzuhalten, dass es erstmalig gelungen ist, aktuelle Diagnosezahlen von Kindern und Jugendlichen mit lebensbedrohlichen und lebensverkürzenden Erkrankungen in Deutschland zu erfassen sowie eine Prävalenzrange abzubilden. Diese können als Grundlage für weitere Untersuchungen im Versorgungssektor dieser äußerst heterogenen Zielgruppe dienen.

## Footnotes

1 Akronym für *Prä*valenzerhebung von *Ki*n*d*ern und Jugendlichen mit lebensverkürzenden und lebensbedrohlichen Erkrankungen.

2 Die modifizierte Liste ist als Anhang des Forschungsberichtes einzusehen. Dieser kann unter https://edoc.hu-berlin.de/bitstream/handle/18452/25451/PraeKids_Abschlussbericht_2022.pdf?sequence=3&isAllowed=y abgerufen werden. Zugegriffen 24.01.2023.

3 https://bundesgesundheitsministerium.de/themen/krankenversicherung/zahlen-und-fakten-zur-krankenversicherung/mitglieder-und-versicherte.html. Zugegriffen 02.05.2022.

4 Die Prävalenzwerte der diagnostischen Gruppierungen und der weiteren Stratifizierungen nach Alter, Geschlecht und Bundesland finden sich im Forschungsbericht unter https://edoc.hu-berlin.de/handle/18452/25451. Zugegriffen am 24.01.2023.

5 Die zugehörigen Tabellen und Ausführungen finden sich im Forschungsbericht unter https://edoc.hu-berlin.de/handle/18452/25451. Zugegriffen am 24.01.2023.

6 Schraffiert dargestellt in Abb. 4.

7 Nachfolgende Informationen zur Datengrundlage der GKV-SV-Daten zur Erhebung der Diagnoseprävalenz durch den Stabsbereich Vertragsanalyse liegen dem Forschungsteam schriftlich vor.

8 Im Sinne durch ökonomischen Druck in den Kliniken entstehender Fehlanreize zur Dokumentation hoher Behandlungsbedarfe.

9 Die Information zur Anzahl der 0‑ bis 19-jährigen Privatversicherten durch das Wissenschaftliche Institut der PKV liegt dem Forschungsteam schriftlich vor.
